# Species-specific variations in reproductive traits of three yellow catfish species (*Pelteobagrus* spp.) in relation to habitats in the Three Gorges Reservoir, China

**DOI:** 10.1371/journal.pone.0199990

**Published:** 2018-07-16

**Authors:** Chuansong Liao, Sibao Chen, Zhiqiang Guo, Shaowen Ye, Tanglin Zhang, Zhongjie Li, Brian R. Murphy, Jiashou Liu

**Affiliations:** 1 State Key Laboratory of Freshwater Ecology and Biotechnology, Institute of Hydrobiology, Chinese Academy of Sciences, Wuhan, Hubei province, China; 2 University of Chinese Academy of Sciences, Beijing, China; 3 Hainan University, Haikou, Hainan province, China; 4 Virginia Polytechnic Institute and State University, Department of Fish and Wildlife Conservation, Blacksburg, VA, United States of America; Ohio State University, UNITED STATES

## Abstract

The reproductive biology of three yellow catfish congeners was studied in the Three Gorges Reservoir of the Yangtze River, China. We compared reproductive traits among the lentic, transitional, and lotic zones. A total of 4502 individuals of the three species was collected, and the sex ratio, size at 50% maturity, spawning season, fecundity, and egg size were determined. Results showed that populations inhabiting the lotic zone spawned earlier than those inhabiting the lentic zone. For the three species, fecundities were significantly higher for populations in the lotic zone than for those in the lentic and transitional zones (*P* < 0.05). *Pelteobagrus vachelli* (Richardson) and *P*. *fulvidraco* (Richardson) displayed an obvious trade-off between egg size and fecundity, whereas *P*. *nitidus* (Sauvage *et* Dabry) produced the largest eggs in the lotic zone. Sex ratios were significantly different among zones (*P* < 0.05, for each species), but the bias patterns were different. Sizes at 50% maturity of female *P*. *nitidus* and *P*. *vachelli* were the largest in the lotic zone and the smallest in the transitional zone, but was similar among zones for *P*. *fulvidraco*. Overall results suggest that the three yellow catfish species developed different reproductive traits among the three habitats in the TGR, whereas the variations reflected further interspecific differences. Our study indicates the importance of riverine habitats for the conservation of species of fish, even for species such as these eurytopic catfish inhabiting the upper reach of the Yangtze River. This study further suggests that species-specific responses should be considered when evaluating the influences of new hydropower projects, even for such closely related species of fish.

## Introduction

Dams have been built for thousands of years and play important societal roles in irrigation, food production, recreation, flood management, electricity generation, and navigation [1, 2]. Dams drastically alter freshwater ecosystems [3–5]. Damming, particularly for the huge projects, can result in large-scale environmental variation, ecosystem fragmentation, and habitat alteration [2, 6]. For example, former riverine habitats can be transformed into a habitat cascade of the lentic, transitional and lotic habitats associated with different environmental conditions along a reservoir [4]. These environmental variations can further affect fishes and other aquatic organisms [7–9]. For fish, many studies have examined effects of damming on migration, assemblage structure, biodiversity, and spawning grounds for particular species [10–14]. By comparison, relatively few studies have focused on the effects of habitat variations on the reproductive biology of species of fish.

Fishes allocate energy among reproduction, growth, and survival [15–17], and different reproductive traits of life history tactics can be developed based on their strong adaptabilities to the environments under genetic constraints [16, 18, 19]. Fishes can exhibit phenotypic plasticity in the face of environmental changes; while the genetic background generates life-history strategy [20]. Genetically based adaptive change may also be generated from long-term selection [21]. Three end-point life history strategies (i.e., periodic, equilibrium, and opportunist strategies) associated with different environments have been proposed [22]. In some studies, variations have been observed in reproductive traits of species of fish inhabiting different habitats due to damming. For example, fecundities and egg sizes of the ice fish *Neoalanx taihuensis* were different above and below the Three Gorges Dam [23], and the Peixe-Cachorro *Acestrorhynchus pantaneiro* exhibited different sex ratios among the lotic, transitional, and lentic habitats of the Itá and Machadinho reservoirs [24]. These studies were primarily based on a single species, and little is known about species-specific patterns in relation to different habitats within a reservoir, although such phenomenon has been reported [18].

The Three Gorges Reservoir (TGR), which is located in the upper reach of the Yangtze River, is the largest reservoir in the world [25]. The TGR was impounded in 2009, and it has an inundation of 1080 km^2^ and total length of 667 km at the highest water level of 175 m [26]. The project attracted global attention from biodiversity and overall environmental perspectives [27, 28] and since the impoundment, has severely changed the former riverine ecosystem into a habitat cascade with different flow regimes, water quality, and aquatic flora and fauna [8, 27, 29, 30]. With the formation of such a longitudinal gradient of habitats, the TGR is an ideal system in which the responses in reproductive traits of species of fish can be observed [2]. The negative effects of the Three Gorges Dam on spawning grounds and migrations of several species of fish have been reported [31–33], but information regarding the effects of habitat variations on aspects of reproductive biology remains limited in the TGR.

The yellow catfish species of the genus *Pelteobagrus* of the family Bagridae have a wide geographic distribution in China. These species inhabit benthic habitats and primarily feed on aquatic insects, freshwater molluscs, and zooplankton [25, 34]. Three species, *P*. *fulvidraco*, *P*. *vachelli*, and *P*. *nitidus*, are distributed in the TGR [35], and contribute to the TGR fishery, although declines in their stocks have been reported [36, 37]. In previous studies, differences in reproductive traits among yellow catfish species inhabiting different rivers and lakes were examined [34, 38], which indicated that *Pelteobagrus* spp. may be sensitive to habitat variations. As a result of the severe changes in riverine habitats following the impoundment of the TGR, the associated organisms, such as the zoobenthos community, an important food resource for yellow catfish, were also affected [30]. The densities of zoobenthos were 8180 ind./m^2^, 184 ind./m^2^, and 243 ind./m^2^ in the lotic, transitional, and lentic zones, respectively [30]. We hypothesized that such large-scale habitat transformations would induce responses in the reproductive biology of these yellow catfish in the TGR. Perera et al. [25] found that *P*. *fulvidraco* exhibited differences in size distribution and gonadosomatic index among different zones of the TGR, and a more comprehensive study based on the three congeneric species would be useful for exploring the potential interspecific difference.

The objective of this study was to analyze the responses in reproductive traits of the three congeneric yellow catfish species to the environmental changes following damming the Yangtze River Three Gorges, which may dictate development of management strategy. As the lack of data prior to dam construction and the present upper section of the TGR more similar to the original riverine situation preceding impoundment, we conducted comparisons among the lower, middle, and upper sections, representing the lentic, transitional, and lotic habitats. We also tested whether interspecific differences in spatial variations of reproductive traits could be observed among these congeneric species.

## Materials and methods

### Ethic statement

Data were collected by all authors in a collaborative effort. All the procedures described in this study were approved by the ethical committee of the Institute of Hydrobiology Chinese Academy of Sciences, Hubei Province, China. All procedures performed in studies involving animals were in accordance with ethical standards (Guidance options on experimental animals) of the Ministry of Science and Technology of the People’s Republic of China. Sampling permits for each location were issued by the China Three Gorges Corporation. The study did not involve any endangered or protected species. All surgery was performed under MS-222 anesthesia, and all efforts were made to minimize suffering.

### Study areas

**Fig 1:**
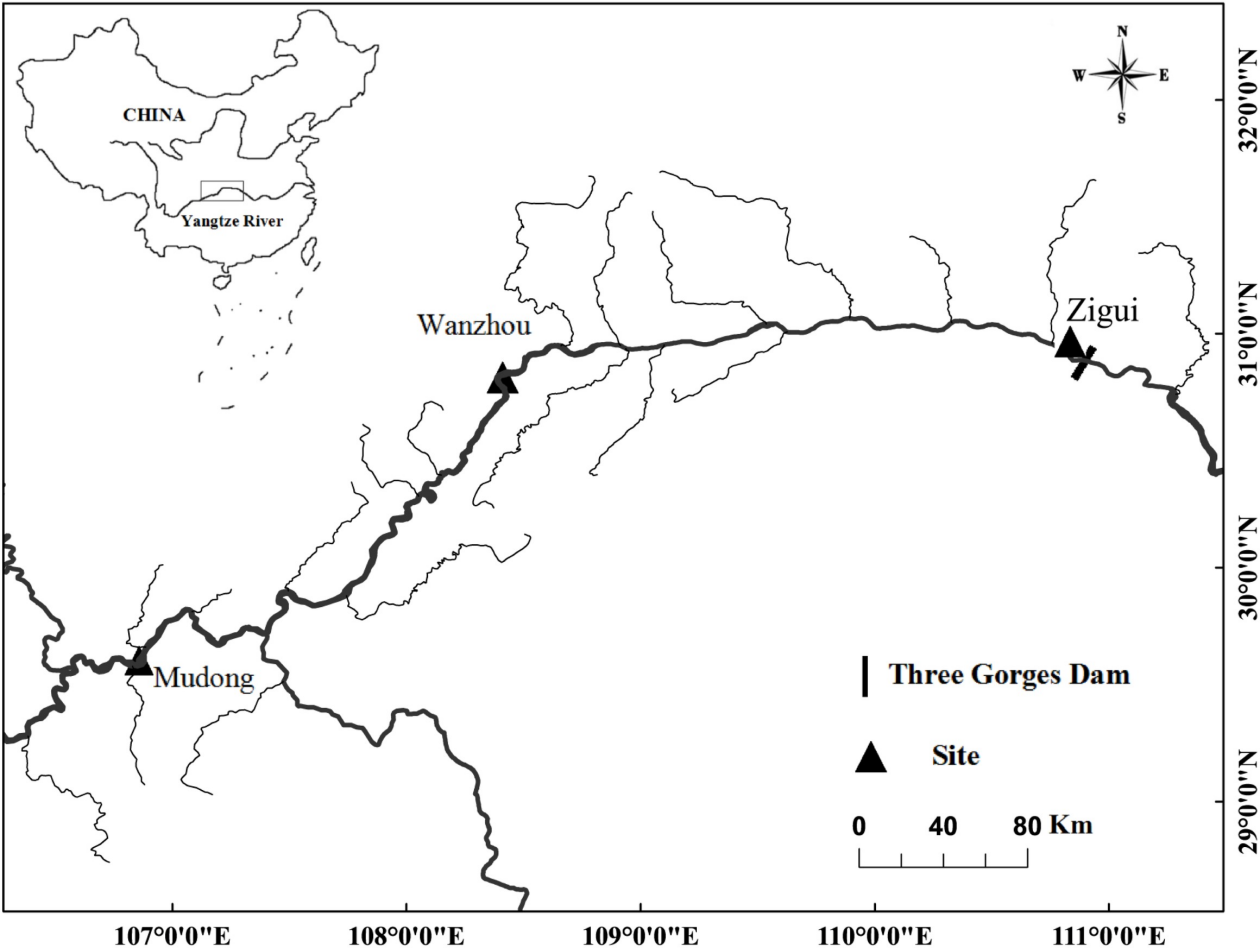
Map of study area along the Three Gorges Reservoir, upper reaches of the Yangtze River. Small solid rectangular shows the location of the Three Gorges Dam. Solid triangles represent the lentic (Zigui), transitional (Wanzhou), and lotic zones (Mudong) of the Three Gorges Reservoir.

We sampled three sections of the TGR: Zigui, Wanzhou, and Mudong. Each section represents a specific habitat type. Zigui (30°51^**′**^36.6^**″**^ N, 110°59^**′**^51.9^**″**^ E) is close to the dam and represents the lentic zone. Wanzhou (30°49^**′**^46.9^**″**^ N, 108°25^**′**^03.8^**″**^ E) is in the middle section (approximately 300 km upstream of the dam), representing the transitional zone with riverine state at low water level but lacustrine state at high water level with the mean annual water flow velocity of 0.26 m/s. Mudong (29°34^**′**^51.4^**″**^ N; 106°51^**′**^01.2^**″**^ E) is in the upper section (approximately 600 km upstream of the dam) and maintains a lotic habitat all year with a mean annual water flow velocity of 1.28 m/s [37] (Fig 1).

During the sampling period, surface water temperatures were recorded using an American HOBO Water Temperature Pro v2 subaqueous temperature collector 1 m below the water level in each zone.

### Fish sampling and data collection

The fish *P*. *fulvidraco*, *P*. *vachelli*, and *P*. *nitidus* were collected in monthly samples from August 2014 to October 2015. To increase the sample size, samples were also collected seasonally from January 2016 to July 2017. *Pelteobagrus fulvidraco* was not observed and therefore was not collected in the transitional zone and was not sampled monthly in the lotic zone because of its rare distribution. Fishes were primarily collected using set gill nets, benthic fyke nets, and lift nets in the lentic zone; benthic fyke nets, set gill nets, and drifting gill nets in the transitional zone; and trawl nets and drifting gill nets in the lotic zone.

All specimens were sorted and identified, and we measured the total body length (L_T_) and body weight (W_B_) of each individual to the nearest 0.01 mm and 0.01 g, respectively. The details of samples for each species in each zone are given in Table 1. Individuals were dissected, and their sexes were differentiated based on the internal anatomy, with the ovaries and testes appearing cylindrical and dendritic, respectively [39]. Six developmental stages of ovaries were assessed by visual inspection [40]. A total of 4502 individuals was dissected, whereas 334 fish could not be sexed. Gonads were separated and weighed to the nearest 0.01 g (W_G_). Eviscerated body weights (W_E_) were measured to 0.01 g. To estimate the absolute and relative fecundity, approximately 1 g of mature ovaries (Stages IV and V) was removed from the anterior, middle, and posterior sections of each lobe, then weighed (W_S_) and fixed in 10% formalin solution. The numbers of oocytes (N_S_) were later counted for each sample.

**Table 1:** Relevant details of numbers (N) of the three yellow catfish species sampled and their mean total length (TL) (mm) and mean body weight (BW) (g) in the lotic, transitional, and lentic zones of the Three Gorges Reservoir.

Sections	*P*. *fulvidraco*			*P*. *vachelli*			*P*. *nitidus*		
	N	TL (Mean ± S.E.)	BW (Mean ± S.E.)	N	TL (Mean ± S.E.)	BW (Mean ± S.E.)	N	TL (Mean ± S.E.)	BW (Mean ± S.E.)
Lotic	253	171.07 ± 2.24	61.83 ± 2.14	682	142.77 ± 2.43	43.59 ± 2.54	831	111.82 ± 0.87	12.47 ± 0.26
Transitional	-	-	-	304	161.46 ± 2.72	60.87 ± 4.53	628	108.87 ± 1.02	10.02 ± 0.24
Lentic	423	148.24 ± 2.77	46.89 ± 2.04	733	142.69 ± 2.7	53.12 ± 2.59	648	95.83 ± 1.01	8.57 ± 0.27

Oocytes of Stage V ovaries that outflowed to enterocoelia were regarded as mature eggs. Mature eggs were photographed with a Leica camera (D.F.C295) connected to a microscope (Leica S8APO) in the laboratory. The diameters of mature eggs (D_M_) were calculated based on the mean length (D_L_) and width (D_W_) diameters measured by ToupView software (Version 3.2). Overall sex ratio (female/male) was determined for each population.

### Data analyses

Chi-square tests (*χ*^2^-test) were used to investigate differences in sex ratios among zones, and the deviations of sex ratios from the expected 1:1 ratio were also determined by *χ*^2^-tests [41, 42]. Logistic regression models were used to determine the size at 50% maturity of females (L_M_). Log-likelihood ratio tests were used to compare the L_M_ among zones for each species.

The gonadosomatic index (% GSI) of females was calculated using the ratio W_G_/W_E_. Spawning season and intensity of reproductive activities were determined based on monthly changes in the GSI. Mann-Whitney *U*-tests were used to compare the GSI of females among zones in April for each species.

Absolute fecundity (F_A_) and relative fecundity (F_R_) were calculated by F_A_ = N_S_W_G_W_S_^-1^ and F_R_ = F_A_W_B_^-1^, respectively. Statistical relationships between the F_A_ and L_T_ were determined. Analyses of covariance (ANCOVA) were used to compare the F_A_ values among populations inhabiting different zones for each species. Analyses of variance (ANOVA) were used to compare the F_R_ values among sections for *P*. *vachelli* and *P*. *nitidus*, and post hoc Tukey-Kramer honestly significant difference (HSD) tests were used for pairwise comparisons. Independent-samples *t*-test was used to compare the F_R_ of *P*. *fulvidraco* between the lotic and lentic zones. Kruskal-Wallis tests were used to assess the differences in the diameters of mature eggs (D_M_) among sections for *P*. *vachelli* and *P*. *nitidus*, whereas for *P*. *fulvidraco*, the difference in the D_M_ between the lotic and lentic zones was tested by a Mann-Whitney *U*-test.

Data normality and homogeneity were evaluated by Kolmogorov-Smirnov tests and Levene^’^s tests, respectively. All statistical analyses were conducted with SPSS Statistics 22.0 (SPSS Inc., Chicago, IL, USA), and differences were considered significant at an alpha level of 0.05. Figures were produced with Sigmaplot 10.0 software.

## Results

### Water temperature

**Fig 2:**
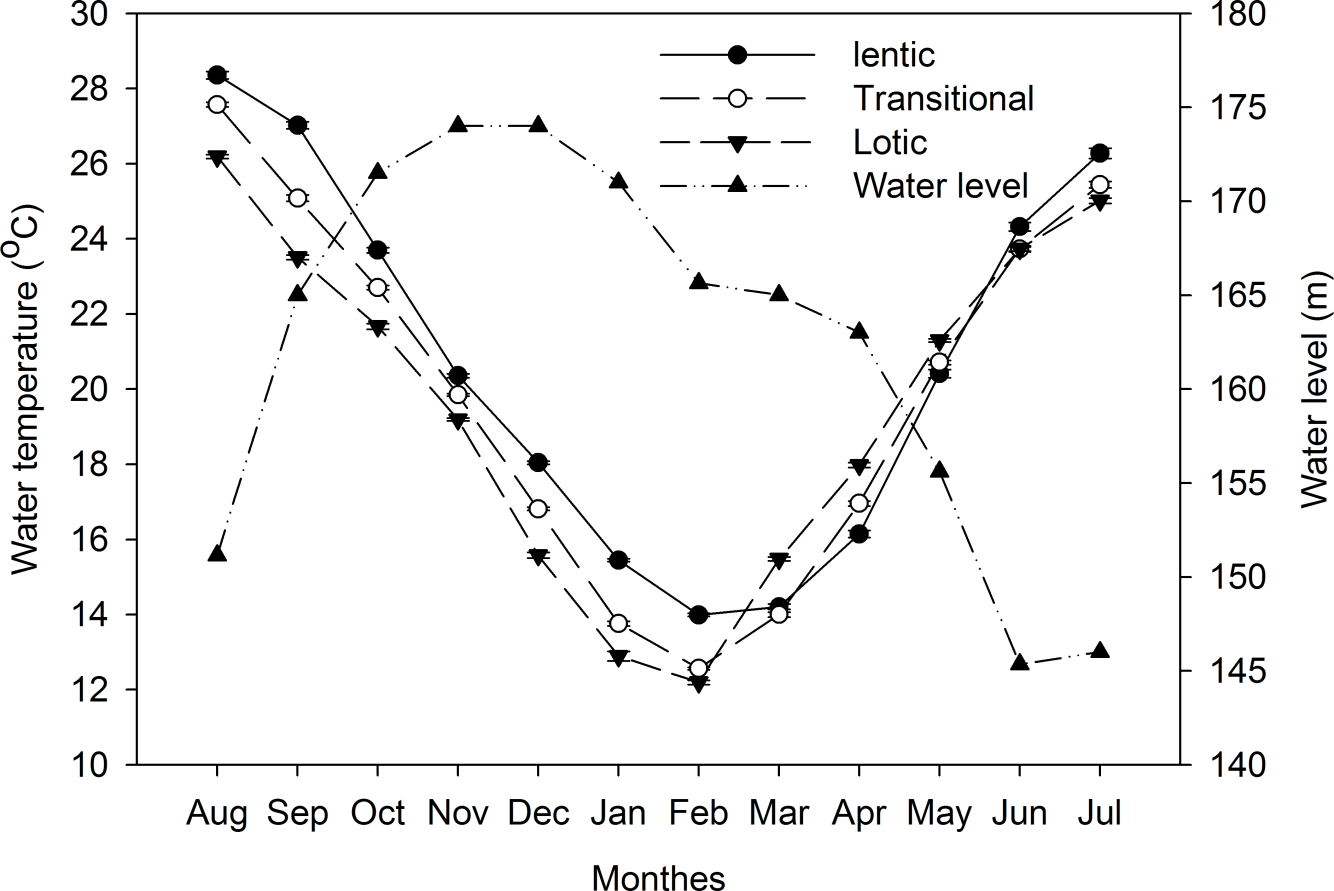
Monthly changes in water temperatures (°C) (mean ± S.E.) and water levels (m) in the Three Gorges Reservoir. Data were collected from August 2014 to July 2015.

The highest mean water temperature was observed in August, and the lowest occurred in February. Water temperature decreased from the lotic to lentic zones between March and June and displayed an opposite trend between July and February (Fig 2). The annual average water temperatures were 20.4 ± 0.08, 19.95 ± 0.07, and 19.59 ± 0.08°C in the lentic, transitional, and lotic zones, respectively.

### Sex ratio

*Pelteobagrus fulvidraco* exhibited different sex ratios between the lentic and lotic zones (*χ*^2^-test: *χ*^2^ = 4.38, *P* = 0.036). Chi-square tests also revealed significantly different sex ratios among the three zones for *P*. *vachelli* (*χ*^2^ = 7.36, *P* = 0.025) and *P*. *nitidus* (*χ*^2^ = 16.16, *P* < 0.05). Specifically, the sex ratios were significantly biased toward males for *P*. *fulvidraco* in the lotic zone (*χ*^2^ = 17.88, *P* < 0.05) but not in the lentic zone (*χ*^2^ = 2.02, *P* = 0.156). For *P*. *vachelli*, the sex ratios were significantly biased toward females in the lentic (*χ*^2^ = 4.18, *P* = 0.04) and lotic zones (*χ*^2^ = 4.93, *P* = 0.026) but not in the transitional zone (*χ*^2^ = 0.44, *P* = 0.506), whereas the ratios of *P*. *nitidus* were significantly biased toward females in the lentic zone (*χ*^2^ = 11.89, *P* < 0.05) but not in the transitional (*χ*^2^ = 0.49, *P* = 0.484) and lotic zones (*χ*^2^ = 0.09, *P* = 0.764) (Table 2).

**Table 2:** Summary findings on reproductive traits of populations of the three yellow catfish species in the lotic, transitional, and lentic zones of the Three Gorges Reservoir.

Species/ Parameter	Zones		
***P*. *fulvidraco***	**Lotic**	**Transitional**	**Lentic**
Sex ratio (female / male)	0.58**^, a^ (n = 251)	Np ^†^	0.82 ^b^ (n = 398)
Size at 50% maturity of females (mm)	119.49 ^a^	Np ^†^	108.6 ^a^
Absolute fecundity (no. of eggs)	5819 ± 630 ^a^ (n = 36)	Np ^†^	3783 ± 334 ^b^ (n = 54)
Relative fecundity (eggs g^-1^)	101.93 ± 4.6 ^a^ (n = 36)	Np ^†^	80.64 ± 3.12 ^b^ (n = 54)
Egg size (mm)	1.706 ± 0.005 ^a^ (n = 540)	Np ^†^	1.725 ± 0.005 ^b^ (n = 953)
Spawning season	Apr to Aug	Np ^†^	May to Sep
***P*. *vachelli***			
Sex ratio (female / male)	1.28*^, a^ (n = 635)	0.9 ^b^ (n = 290)	1.27*^, a^ (n = 605)
Size at 50% maturity of females (mm)	171.18 ^a^	143.02 ^b^	150.78 ^c^
Absolute fecundity (no. of eggs)	6510 ± 474 ^a^ (n = 48)	3496 ± 366 ^b^ (n = 46)	5994 ± 531^b^ (n = 56)
Relative fecundity (eggs g^-1^)	76.82 ± 2.99 ^a^ (n = 48)	62.15 ± 2.05 ^b^ (n = 46)	65.69 ± 2.11 ^b^ (n = 56)
Egg size (mm)	1.818 ± 0.004 ^a^ (n = 805)	1.918 ± 0.006 ^b^ (n = 872)	1.889 ± 0.004 ^c^ (n = 1484)
Spawning season	Apr to Aug	Apr to Aug	May to Sep
***P*. *nitidus***			
Sex ratio (female / male)	0.97 ^a^ (n = 800)	1.09 ^a^ (n = 588)	1.49 **^, b^ (n = 601)
Size at 50% maturity of females (mm)	112.31 ^a^	101.84 ^b^	111.16 ^a^
Absolute fecundity (no. of eggs)	1111 ± 86 ^a^ (n = 52)	719 ± 29 ^b^ (n = 50)	930 ± 64 ^b^ (n = 60)
Relative fecundity (eggs g^-1^)	55.10 ± 2.38 ^a^ (n = 52)	41.73 ± 1.27 ^b^ (n = 50)	45.36 ± 1.63 ^b^ (n = 60)
Egg size (mm)	2.116 ± 0.004 ^a^ (n = 1083)	2.056 ± 0.005 ^b^ (n = 1251)	1.898 ± 0.004 ^c^ (n = 748)
Spawning season	Apr to Aug	Apr to Aug	May to Sep

† Np: not present.

Values of absolute fecundity, relative fecundity, and egg size are mean ± S.E.

Where relevant the values in parentheses indicate the number sampled.

*, sex ratio was significantly different with 1:1 at *P* < 0.05

**, sex ratio was highly significantly different with 1:1 at *P* < 0.001.

For any one parameter the values with the same superscript are not significantly different (*P* > 0.05).

### Size at 50% maturity

The sizes at 50% maturity of females (L_M_) were not significantly different between the lotic and lentic zones for *P*. *fulvidraco* (Likelihood ratio test: *χ*^2^ = 0.692, *P* = 0.406), whereas the L_M_ of *P*. *vachelli* was significantly larger in the lotic zone than in the lentic zone, with the smallest L_M_ in the transitional zone (*χ*^2^ = 25.779, *P* < 0.05). *Pelteobagrus nitidus* also exhibited the smallest L_M_ in the transitional zone (*χ*^2^ = 13.183, *P* = 0.001), but the L_M_ was similar between the lotic and lentic zones (*χ*^2^ = 0.967, *P* = 0.325) (Table 2).

### Spawning season

**Fig 3:**
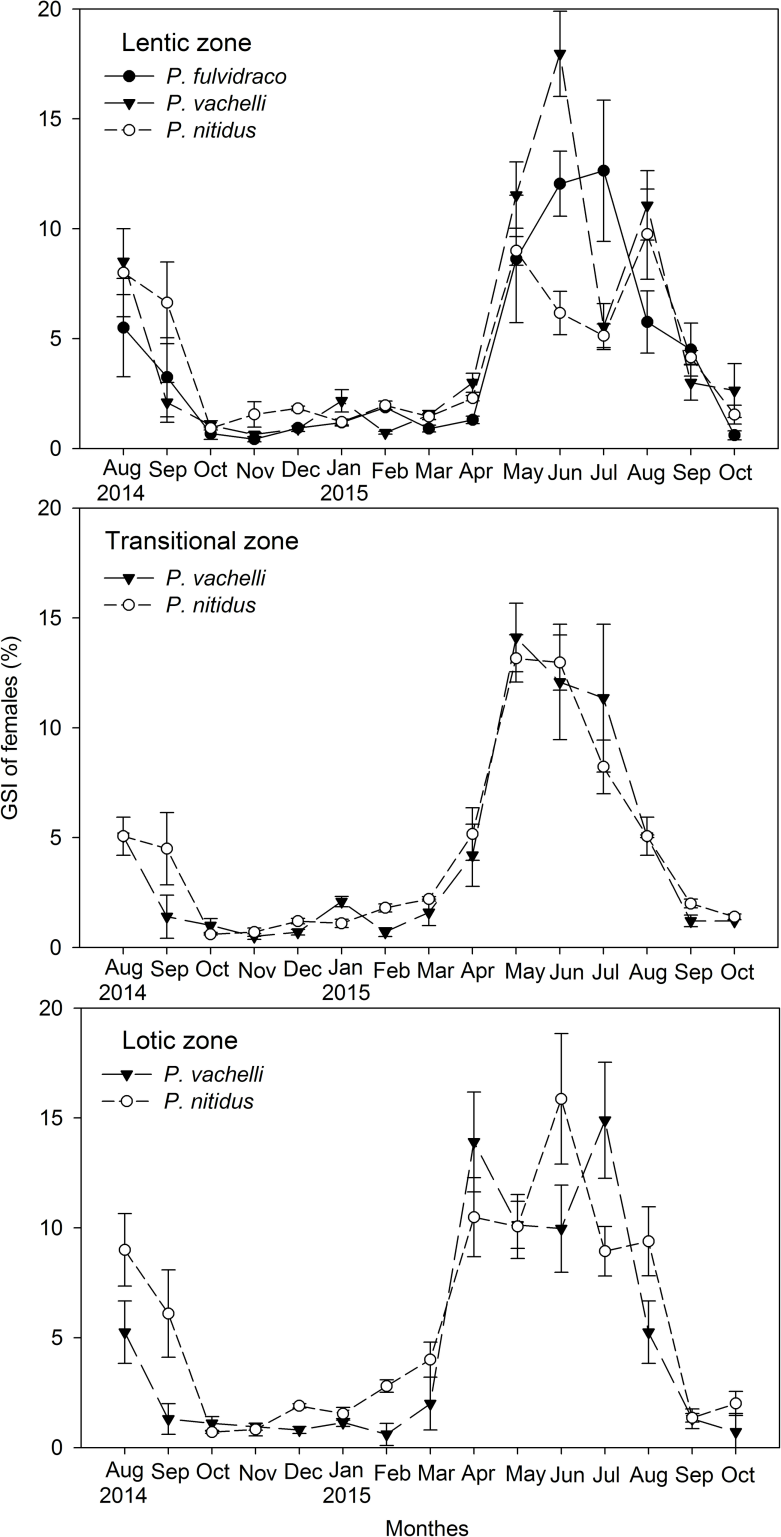
Monthly changes of gonado-somatic indexes (GSI) (mean ± S.E.) of females. Data were collected from August 2014 to October 2015.

The three species spawned earlier in the lotic and transitional zones than in the lentic zone (Table 2). The GSI of females considerably increased in May for populations in the lentic zone, whereas the increase occurred in April for those in the lotic and transitional zones (Fig 3). In April, the GSI was significantly higher in the lotic zone than in the lentic zone for *P*. *fulvidraco*, *P*. *vachelli*, and *P*. *nitidus* (Mann-Whitney *U*-test: *W* = 210, *P* < 0.05; *W* = 1683, *P* < 0.05; and *W* = 2823, *P* < 0.05, respectively).

The three species displayed staggered spawning peaks in each zone. In the lentic zone, the GSIs of females were observed as peaks in July for *P*. *fulvidraco*, in June for *P*. *vachelli*, and in August for *P*. *nitidus*. In the lotic zone, the GSI values of *P*. *vachelli* and *P*. *nitidus* were observed as peaks in July and June, respectively. The GSI declined to the lowest level in October for populations in the lentic zone, whereas the lowest levels were reached in September for populations in the lotic and transitional zones; the GSI then remained at low levels until the next spawning season (Fig 3).

### Fecundity

**Fig 4:**
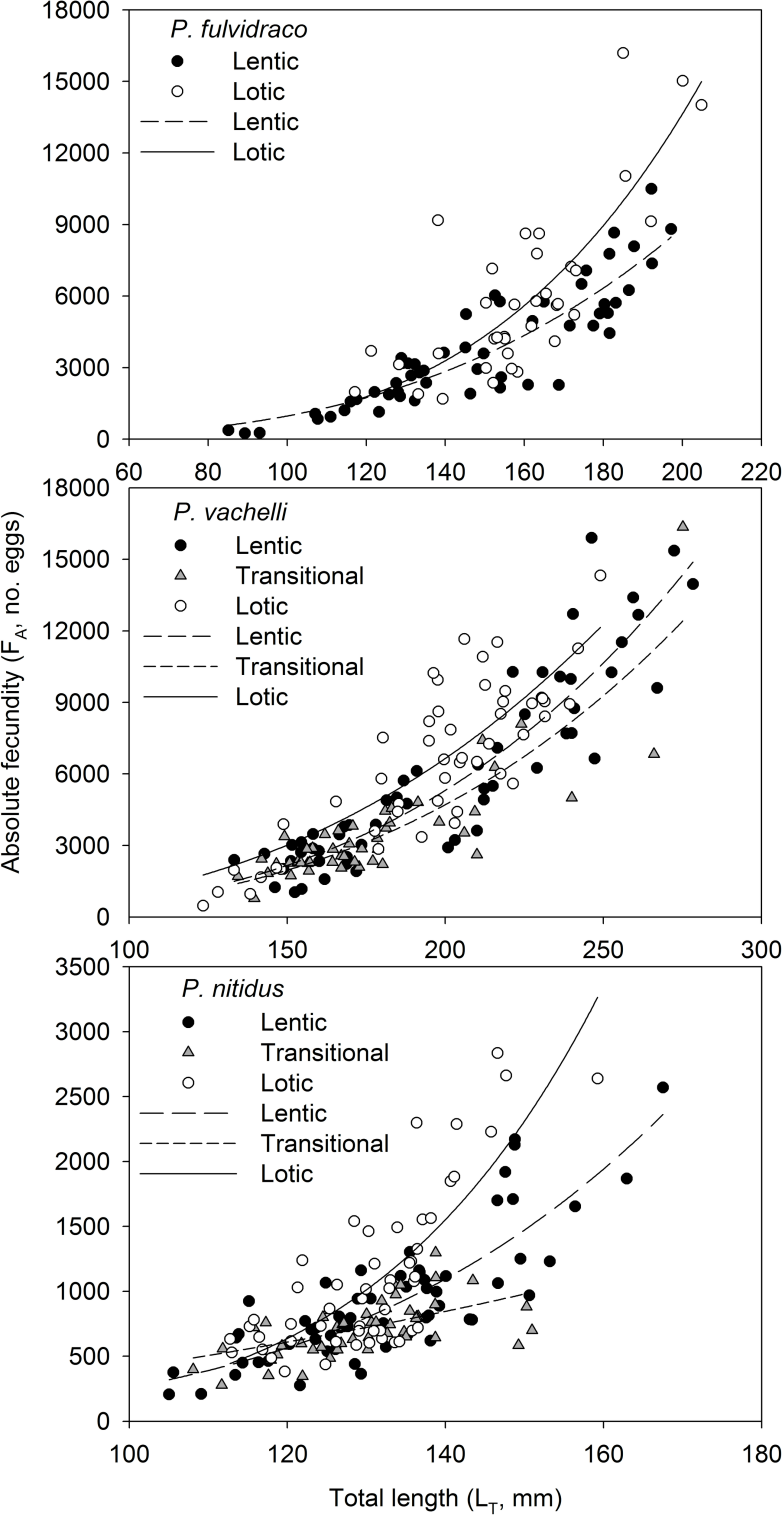
Relationships between absolute fecundity (F_A_) and total length (L_T_) of the three yellow catfish species in the Three Gorges Reservoir.

The fecundities of the three species were the highest in the lotic zone of the TGR (Table 2). The absolute fecundity (F_A_) increased with increasing total body length (L_T_) (Fig 4). The F_A_ was significantly different between the lotic and lentic zones for *P*. *fulvidraco* (ANCOVA: *F*_1, 88_ = 9.661, *P* = 0.003) and was also significantly different across zones for *P*. *vachelli* (*F*_2, 147_ = 6.857, *P* = 0.001) and *P*. *nitidus* (*F*_2, 159_ = 13.866, *P* < 0.05) (Table 2).

The relative fecundities (F_R_) of *P*. *fulvidraco* were significantly higher in the lotic zone than in the lentic zone (Independent-samples *t-*test: *t* = -4.49, *P* < 0.05) and were also significantly different among zones for *P*. *vachelli* (ANOVA: *F*_2, 147_ = 7.275, *P* = 0.001) and *P*. *nitidus* (*F*_2, 159_ = 12.386, *P* < 0.05). Specifically, the F_R_ was significantly higher in the lotic zone than in the transitional (HSD test: *P =* 0.001; *P* < 0.05) and lentic zones (*P* = 0.016; *P* = 0.001) but was similar between the lentic and transitional zones (*P* = 0.552; *P* = 0.407) for both *P*. *vachelli* and *P*. *nitidus* (Table 2).

### Egg size

The mean egg diameters (D_M_) were negatively correlated with the F_R_ for *P*. *vachelli* and *P*. *fulvidraco*. Specifically, *P*. *fulvidraco* produced larger eggs in the lentic zone than in the lotic zone (Mann-Whitney *U*-test: *W* = 367448, *P* < 0.05). For *P*. *vachelli*, the D_M_ was significantly larger in the transitional zone than in the lentic zone and was the smallest in the lotic zone (Kruskal-Wallis test: *H* = 299.36, *P* < 0.05). The D_M_ of *P*. *nitidus* was the largest in the lotic zone, followed by the transitional and lentic zones (*H* = 475.62, *P* < 0.05) (Table 2).

## Discussion

Various investigators reported that reproductive traits of some species of fish vary with different environments [18, 21, 43, 44]. In this study, large differences were observed across the three zones in the TGR for key reproductive traits, including the sex ratio, size at 50% maturity, spawning season, fecundity, and egg size of the three yellow catfish species. The three zones from which the yellow catfish samples were derived represent distinct habitats: lotic, transitional, and lentic, following damming the Yangtze River Three Gorges. The three yellow catfish species are not migratory fishes [45], and the migratory would be unlikely to be the main factors for these variations.

Variations in life-history traits can be generated by genetics or environment, or combinations of both [19, 46]. For our study, the question of whether the phenotypic variations in reproductive traits of the three yellow catfish species are based on genetic differences or phenotypic plasticity remains uncertain. It is still uncertain whether there are genetic differences among populations in the lentic, transitional, and lotic zones within the TGR, but the degree to which all reproductive traits (sex ratio, size at maturity, spawning season, fecundity, and egg size) varied suggests that genetic differences may exist. The TGR is located in the upper section of the Yangtze River. Wang et al. [47] found that populations of *Pelteobagrus fulvidraco* and *P*. *vachelli* in the upstream reaches of the Yangtze River were genetically divergent from populations in the mid- and down-stream reaches, and attributed the divergence to natural Three Gorges influences and the anthropogenic influences of the Gezhouba and the Three Gorges dams. Our study supported such prediction [47], and found different fecundities between populations of *P*. *vachelli* from the lentic zone of the TGR and middle stream of the Yangtze River [48], which indicated that the genetic divergence may exist because of the blocking of genetic exchange by the dams. The fecundities, however, were similar between the lentic and transitional zones within the TGR which are separated by the natural Three Gorges, which may indicate less genetic divergence due to the barrier of the Three Gorges than the more-extreme barriers presented by the dams. Meanwhile, large difference was observed between the lotic and transitional zones, which are both located in the upper section of the natural Three Gorges. Due to the aforementioned facts, we deduced that genetic differentiation may exist among populations inhabiting the lentic, transitional, and lotic zones, but different environments also significantly contributed to different reproductive traits. Such combined effects have also been reported in other studies [46, 49]. Further research quantifying genetic differences among catfish species in the three zones within the TGR would be useful in disentangling these co-varying effects.

The three yellow catfish species exhibited a general tendency for the highest fecundity in populations inhabiting the lotic zone of the TGR, which is the habitat that most closely represents the original status of the river ecosystem. We also noted that the fecundity of *P*. *vachelli* in the TGR was lower than that in the Jialing River, which is a tributary located in the upper reach of the Yangtze River with a more typical riverine state [50]. Moreover, fecundities of populations of *P*. *fulvidraco* and *P*. *vachelli* that inhabit the lotic habitat of the TGR were greater than those of populations in the Dongting Lake and the Ce Lake [34, 38]. These results indicated that habitat conditions play an important role in fecundity of yellow catfish species and that transition to the lentic and transitional habitats has negative effects on fecundity after impoundment. Similar findings have also been observed in other studies. For example, *Geophagus brasiliensis* exhibited higher fecundity and gonadosomatic indexes in riverine habitats than in lacustrine habitats [43]. Additionally, Godinho et al. [44] found that lotic fishes exhibited higher relative fecundity than lentic fishes in Brazil. The diversification observed in the present study is consistent with the three end-points of life history strategies [22]. The lentic zone is a more stable environment than the lotic and transitional zones in the TGR, suggesting that yellow catfish populations inhabiting the lentic habitat are likely approaching an equilibrium strategy with reduced fecundity. Populations inhabiting the lotic habitat exhibit a periodic strategy with the highest fecundity and late maturity, whereas populations inhabiting the transitional habitat tend to adopt an opportunistic strategy with the lowest fecundity and the earliest maturity [17, 22].

Food availability can also affect fecundity, and because reproduction requires abundant resources, a high level of food intake can lead to high fecundity by positively affecting body size [16]. In the TGR, the density of zoobenthos in the lotic zone is significantly higher than that in the transitional and lentic zones [30], which is consistent with the spatial variations in fecundity. High density of zoobenthos can increase food consumptions for yellow catfishes, which commonly feed on zoobenthos, such as aquatic insects [38]. We found that the three species consumed a higher proportion of zoobenthos in the lotic zone than in the lentic zone (S1 Fig). Additionally, we found that the hepatosomatic indexes were higher in the lotic zone than in the lentic and transitional zones (S2 Fig), indicating better feeding conditions in the lotic zone to some degree [51]. Abundant food availability at a suitable time can indirectly lead to high fecundity [16, 52]. The spatial variations of fecundity of the three yellow catfish species are consistent with these predictions, which suggested that the density of zoobenthos should be considered an important factor in explaining the variations.

In the present study, the yellow catfish populations inhabiting the lotic zone showed another general pattern of earlier onset of spawning than those in the other two zones. The reproductive cycle of fishes primarily rely on temperature and photoperiod [16, 53]. Water temperature can significantly affect breeding processes [53, 54] and usually exhibits spatial variations within an aquatic ecosystem [54, 55]. In the TGR, the water temperature decreases from the lotic to the lentic zones between March and June, indicating that the water temperature is a key cue for the spawning onset of the three species given the similar photoperiod conditions among the three zones at similar latitude. Additionally, *P*. *fulvidraco* spawned from March in the Zhujiang River located in Guangdong Province, South China, and from June in the Tanghe Reservoir located in Liaoning Province, North China [56, 57]. The findings further illustrated the effect of water temperature on spawning cycles.

Water temperature also affects fecundity and egg size [58, 59], and some species of fish tend to exhibit low fecundity in cold environments [54, 60]. Our results were inconsistent with those observations, as the highest fecundity was observed in the lotic zone with the lowest water temperature. Jigyasu and Singh [61] also found a negative correlation between water temperature and fecundity for a snail species *Lymnaea acuminate*. Compared with the relatively homogeneous habitat of lakes, such as that of Erhai Lake [54], the three zones of the TGR are much more heterogeneous environments, but the mean annual water temperature in the lentic zone is higher than that in the transitional and lotic zones by only 0.45°C and 0.81°C, respectively. It seems unlikely that the temperature difference was large enough to explain the fecundity variants of the three species, but the significantly different habitats with large differences in food availability can sufficiently develop these differences. Similarly, Thorsen et al. [62] observed different fecundities for the Atlantic cod *Gadus morhua* among four different seas along a latitudinal gradient and concluded that the difference could not only be explained by different temperatures but also from fishing pressure and food availability.

In the present study, the sex ratio, size at 50% maturity, and egg size did not exhibit consistent pattern of spatial variation for the three species, indicating species-specific responses in reproductive traits for these related species. In general, to increases the survival of given eggs, a trade-off usually occurs between egg size and fecundity [63, 64], such as that observed for *P*. *fulvidraco* and *P*. *vachelli* in this study. *Pelteobagrus nitidus* inhabiting the lotic zone displayed the highest fecundity and the largest egg size, which indicated a high reproductive investment [18]. A positive correlation between egg size and fecundity was also reported in Thorpe et al. [65]. Additionally, we found that the GSI values of mature ovaries in the lotic zone (14.43%) was higher than that in the lentic (9.96%) and transitional zones (13.01%) for *P*. *nitidus*, which was further evidence of the high reproductive effort in the lotic zone [66].

Sex of yellow catfish is determined genetically by an XY system [67]. As for environmental factors, researchers found that sex ratios of *P*. *vachelli* showed no difference with a 1:1 ratio observed at temperatures of 20 and 24°C [41]. Zhang et al. [42] found that *P*. *fulvidraco* and *P*. *vachelli* displayed no temperature-dependent sex determination at water temperature of 26, 29, 32, and 34°C. Nevertheless, the water temperature would be unlikely to affect sex differentiation of these species during the current range of water temperature in the TGR. Studies in other areas, such as the Jialing River, middle reach of the Yangtze River, and the Ce Lake, also found no difference from a 1:1 ratio for these species [34, 48, 50]. Other factors such as pH, density or social status have not been reported to affect sex determination for these species [68, 69], and pH was similar among the three zones, with 7.64, 7.67, and 7.65 in the lentic, transitional, and lotic zones, respectively. Due to the aforementioned facts, the spatial variations and the species-specific variation patterns in sex ratio of the three catfish species may be related to other factors such as fishing activity [70]. On the other hand, the largest L_M_ values of *P*. *vachelli* and *P*. *nitidus* were observed in the lotic habitat with the lowest water temperature, which was confirmed by other studies [54, 71], but the smallest L_M_ was observed in the transitional habitat with a moderate water temperature. These results provided further indication of the opportunistic strategy for populations inhabiting the transitional zone of the TGR [22]. For *P*. *fulvidraco*, the L_M_ values were similar between the lotic and lentic zones, suggesting that other factors and species-specific habitat requirements were also relevant [17, 18, 72, 73].

Hypotheses such as the phylogenetic, trophic, and competition hypotheses have been proposed to explain species-specific variations in life history traits [18]. Our results are not consistent with the first two hypotheses, because the three congeneric species with similar feeding habits exhibited interspecific difference in variations to the same habitat variation. We propose that the competition hypothesis is the most reasonable one because of the importance of reducing inter-species competition for these closely related species [72]. The staggered GSI peaks of populations inhabiting each zone also provide support for this hypothesis.

In conclusion, this study demonstrated broad variations in reproductive traits of three congeneric yellow catfish species among three different habitats (i.e., lentic, transitional, and lotic) along the TGR. These results provide evidence for the severe effect of such large-scale hydropower projects on the reproductive biology of these benthivore species of fish by creating significantly different habitats with different food availabilities and water temperatures, among other factors. Moreover, this study emphasizes the importance of lotic habitat for lotic species [33] and eurytopic species, such as these three catfish species. Fortunately, a lotic stretch of more than 300 km and tributaries still remain between the tail of the TGR and the Xiangjiaba Dam [74]. From a management perspective, new hydropower projects should be rejected in this area and better habitat management will be required in the future. The present study suggests that species-specific responses should be considered when evaluating the influences of a new hydropower plant, even for such closely related species of fish such as the studied catfish. The findings may be of importance considering the new projects in the upper Yangtze River basin, such as the Baihetan and the Wudongde Reservoir projects [75].

## Supporting information

S1 Fig Food composition by the three yellow catfish species based on weight data in the lentic and lotic zones of the Three Gorges Reservoir. (TIF)

S2 Fig Hepatosomatic indexes of the three yellow catfish species in the lentic, transitional, and lotic zones of the Three Gorges Reservoir. (A) ZG, WZ, and MD represent the lentic, transitional, and lotic zones. (B) Dashed lines represent mean values.
(TIF)

S1 File Data used to estimate the reproductive traits of the three yellow catfish species in the Three Gorges Reservoir. (XLSX)
