# Supplementary figures and images for: Species-specific variations in reproductive traits of three yellow catfish species (Pelteobagrus spp.) in relation to habitats in the Three Gorges Reservoir, China
Source: PLoS One. 2018 Jul 16;13(7):e0199990. doi: 10.1371/journal.pone.0199990 (PMC6047775; doi:10.1371/journal.pone.0199990)

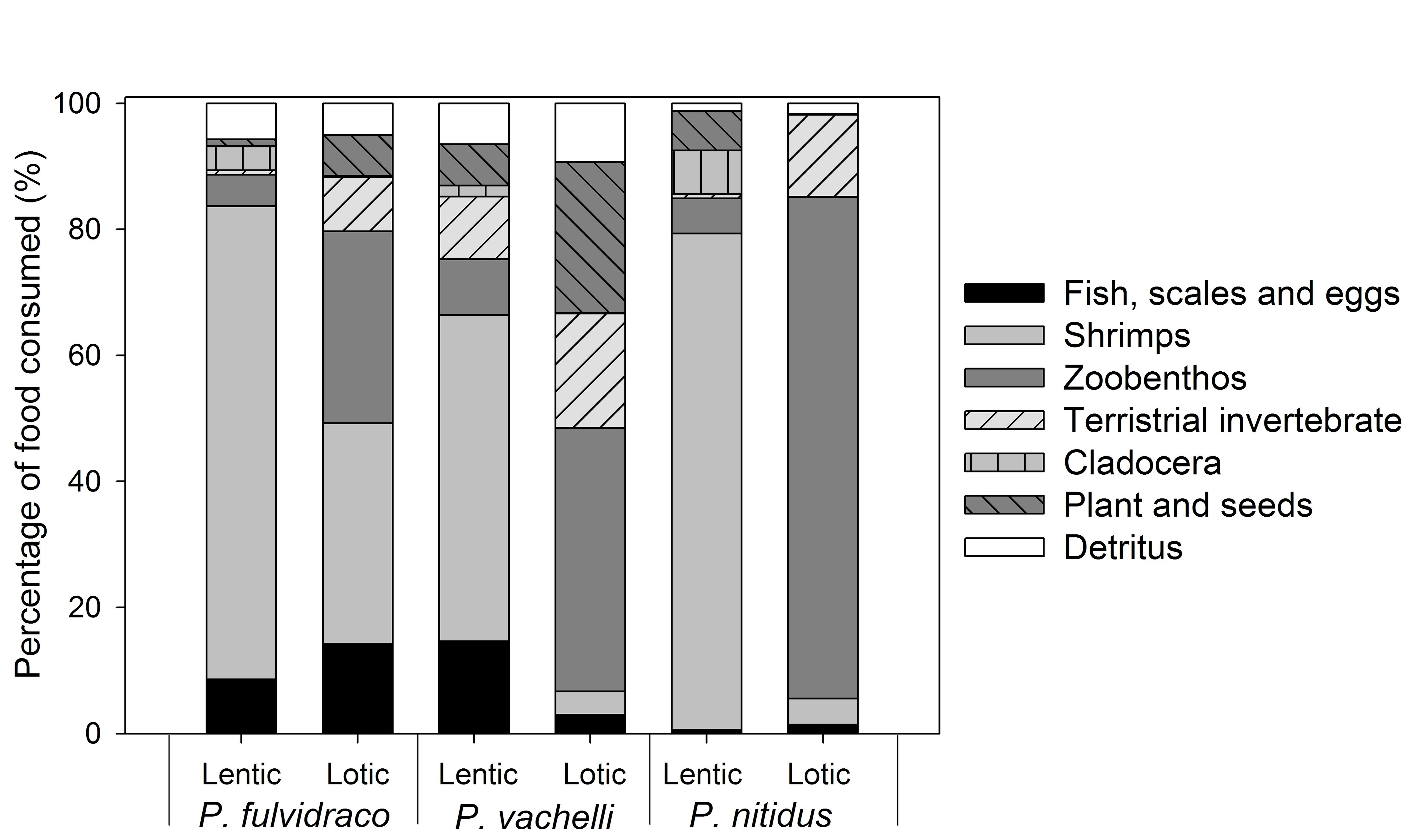

Supplement: S1 Fig — (TIF) [file pone.0199990.s001.tif]

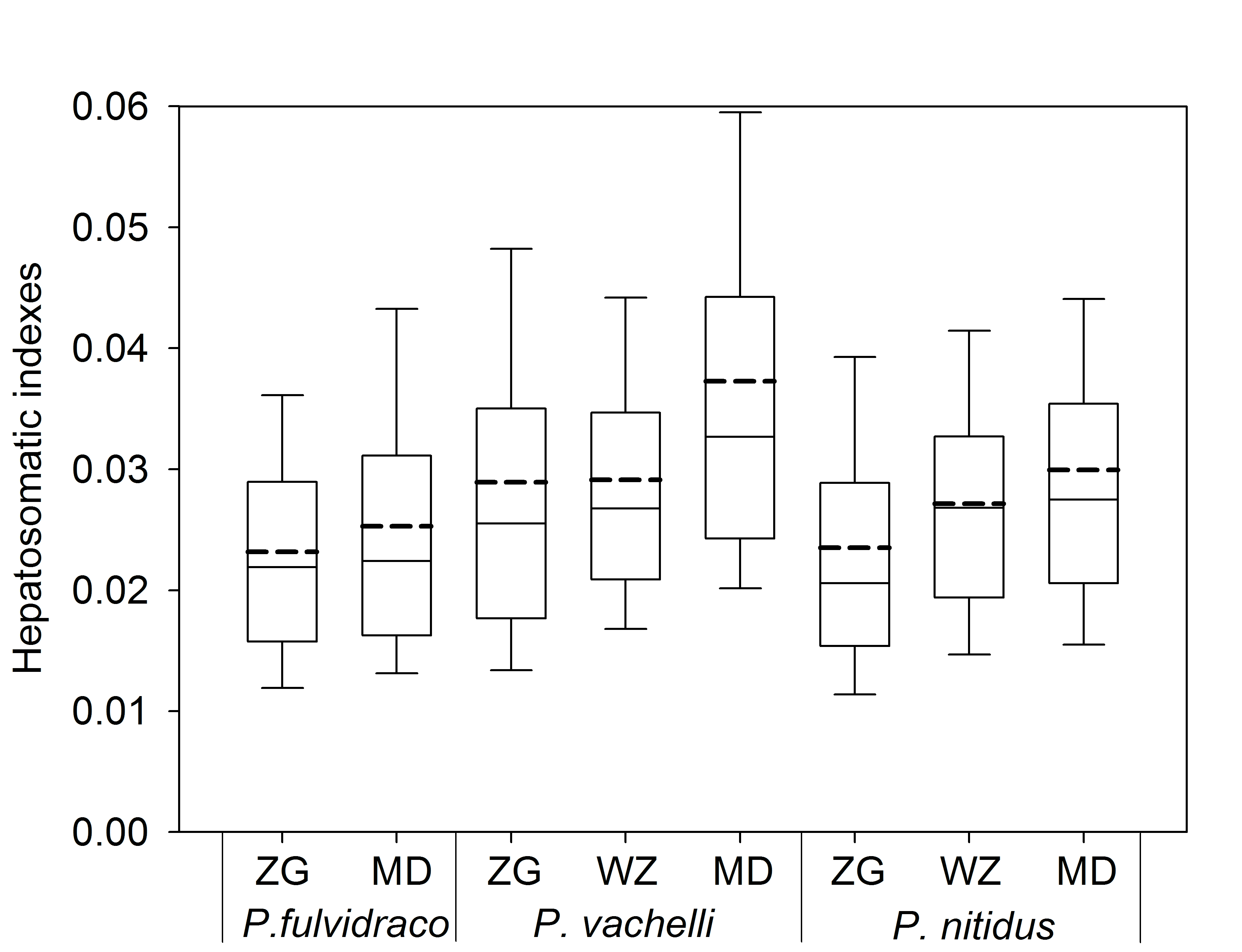

Supplement: S2 Fig — (A) ZG, WZ, and MD represent the lentic, transitional, and lotic zones. (B) Dashed lines represent mean values. (TIF) [file pone.0199990.s002.tif]
